# Effects of a moderate intake of beer on markers of hydration after exercise in the heat: a crossover study

**DOI:** 10.1186/s12970-015-0088-5

**Published:** 2015-06-06

**Authors:** David Jiménez-Pavón, Mónica Sofía Cervantes-Borunda, Ligia Esperanza Díaz, Ascensión Marcos, Manuel J. Castillo

**Affiliations:** Department of Medical Physiology, School of Medicine, University of Granada, Avd. Madrid s/n, CP: 18012 Granada, Spain; GALENO Research Group, Department of Physical Education, Univeristy of Cadiz, Puerto Real, Spain; Immunonutrition Research Group, Department of Metabolism and Nutrition, Institute of Food Science and Technology and Nutrition. Spanish National Research Council (CSIC), Madrid, Spain

**Keywords:** Dehydration, Hot conditions, Running, Alcohol, Diuresis, Young men

## Abstract

**Background:**

Exercise in the heat causes important water and electrolytes losses through perspiration. Optimal rehydration is crucial to facilitate the recuperation process after exercise. The aim of our study was to examine whether a moderate beer intake as part of the rehydration has any negative effect protocol after a short but dehydrating bout of exercise in the heat.

**Methods:**

Sixteen active male (VO_2_max, 56 ± 4 mL/kg/min), were included in a crossover study and performed a dehydrating exercise (≤1 h running, 60 %VO_2_max) twice and 3 weeks apart, in a hot laboratory setting (35 ± 1 °C, humidity 60 ± 2 %). During the two hours following the exercise bouts participants consumed either mineral water *ad-libitum* (W) or up to 660 ml regular beer followed by water *ad-libitum* (BW). Body composition, hematological and serum parameters, fluid balance and urine excretion were assessed before, after exercise and after rehydration.

**Results:**

Body mass (BM) decreased (both ~ 2.4 %) after exercise in both trials. After rehydration, BM and fat free mass significantly increased although BM did not return to baseline levels (BM, 72.6 ± 6.7 to 73.6 ± 6.9; fat free mass, 56.9 ± 4.7 to 57.5 ± 4.5, no differences BW vs W). Beer intake did not adversely affect any measured parameter. Fluid balance and urine excretion values did not differ between the rehydration strategies.

**Conclusions:**

After exercise and subsequent water losses, a moderate beer (regular) intake has no deleterious effects on markers of hydration in active individuals.

**Electronic supplementary material:**

The online version of this article (doi:10.1186/s12970-015-0088-5) contains supplementary material, which is available to authorized users.

## Background

Exercise in the heat causes important water and electrolyte losses through perspiration. Optimal rehydration is crucial to facilitate the recuperation process after exercise [1, 2]. The American College of Sport Medicine (ACSM) [1] guidelines were designed to achieve an optimal rehydration protocol after exercise with sport beverage intake. In general, fluid replacement beverages should contain water and ~20–30 mEq/L sodium (chloride as the anion), ~2–5 mEq/L potassium, and ~5–10 % carbohydrate, depending on the specific exercise task (*e.g.*, intensity and duration) and enviromental conditions [1].

In Western countries it is common practice to drink beer (regular) after exercise [3, 4], as part of social relationships [5] or after other physical activities (ie: manual worker in warm environments) [6]. This is particularly the case in the non-professional (amateur) context, where this practice is considered part of the social aspect of many sport activities. This is mostly the case when temperature conditions, and consequently perspiration rates, are high. Regular beer is a naturally fermented beverage, composed mainly of water, which contains some nutrients (such as carbohydrates, B vitamins, and minerals) [7]. Except for sodium and alcohol, beer has similar properties to that of sports beverages [1]. However, the standard beer (regular) commonly consumed contains alcohol (4–4.5 %) rather than low alcohol beers which are rarely consumed [5] (Table 1). Alcohol may represent a serious drawback that can blunt beer’s rehydrating capacity and negatively affect the restoration of fluid balance increasing the diuretic response in the body [8, 9]. Recently, interest has increased in understanding how alcohol intake affects sports performance [8, 10, 11], but little is known regarding how alcohol influences the recovery process and hydration status after exercise in the heat [12–15]. A moderate intake of alcohol (2–4 drinks/day) has showed to have health benefits [16], however, individuals who regularly consume beer after sport may not have clear information about its dose-response effect. This is an important issue to study in order to advise those who regularly drink beer after sport or physical activity in the heat, or those using this beverage by its well-known thirst quenching effect [17, 18].

**Table 1:** Composition of different beers and Sport Drink (for 100 g of edible portion)

Components	Beer-stout	Regular beer	Beer low alcohol	Sport drink
General composition				
Alcohol (ethanol) (g)	6.15	3.96	3.5	0
Total energy	278 KJ (67 Kcal)	176 KJ (42 Kcal)	141 KJ (34 Kcal)	134 KJ (32 Kcal)
Total fat (total lipids) (g)	trace	0	0	0
Total protein (g)	0.6	0.5	0.2	0
Water (humidity) (g)	88.5	92.4	97.7	92.1
Carbohydrates				
Sugars (g)	5.3	3.1	2.1	7.9
Fiber (total dietary) (g)	0	0	0	0
Fat				
Fatty acids, total	trace	0	0	0
monounsaturated (g)				
Fatty acids, total	trace	0	0	0
polyunsaturated (g)				
Total saturated fatty acids (g)	trace	0	0	0
Cholesterol (mg)	0	0	0	0
Vitamins				
Vitamin A^a^ (μg)	trace	0	trace	0
Vitamin D (μg)	0	0	0	0
Vitamin E^b^ (mg)	0	0	0	0.5
Niacin total equivalents	0.815	0.43	0.7	0.9
(Vitamin B3) (mg)				
Riboflavin (Vitamin B2) (mg)	0.055	0.03	0.02	0
Thiamine (Vitamin B1) (mg)	trace	0	trace	0
Vitamin B12 (μg)	0.28	0.15	trace	0
Vitamin B6 (mg)	0.05	0.06	0.05	0.1
Vitamin C (ascorbic acid) (mg)	0	0	0	0
Minerals				
Calcium (mg)	11	8	10	0.8
Total iron (mg)	0.05	0.01	trace	trace
Potassium (mg)	92	37	34	2.2
Magnesium (mg)	16.5	9.6	6	5
Sodium (mg)	11.5	4.4	7	24
Phosphorous (mg)	33.5	55	20	1
Iodide (μg)	8	8	1	1
Total selenium (μg)	1.2	1.2	trace	trace
Zinc (Cinc) (mg)	0.01	0.01	trace	trace

BEDCA. Base de Datos Española de Composición de Alimentos (online). <http://www.bedca.net>

^a^Equivalents of retinol as retinos and carotenoids activities

^b^Alpha tocpherol equivalents from E vitamers activities

The objective of the present study was to examine whether a moderate beer intake as part of the rehydration has any negative effect after a short but dehydrating bout of exercise in the heat.

## Methods

### Study design

A crossover, counter-balanced and randomized study was conducted in a group of 22 physically active men from the southerm Spain (Granada) to examine the effect of rehydration with beer plus water (intervention phase) *vs.* water alone (control phase) after a running bout under hot environmental laboratory conditions.

The study protocol was performed in accordance with the ethical standards established in the Declaration of Helsinki. The study was approved by the Review Committee for Research Involving Human Subjects of the University of Granada and by the Bioethics Committee of the Spanish National Research Council (CSIC). All participants were informed about the characteristics of the study and signed an informed consent. Inclusion criteria were being physically active, having a good fitness level, following a varied diet that included alcohol in moderate amounts, and being devoid of personal or family history of alcoholism. Participants were considered physically active if they practiced physical activity for at least 1 h, ≥4 times/week. To determine the level of fitness, aerobic capacity was assessed prior to inclusion and participants’ VO_2max_ should be above 50 ml/kg/min to be considered with a “good fitness level”. Maximal aerobic capacity (MAS) and maximal heart rate (Polar® 720i, Polar Electro Inc, Kempele, Finland) were assessed by the Université de Montréal Track Test [19]. A previously validated equation was used to estimate VO_2max_ [20]. A minimum of 40-min of treadmill running was required. Finally, participants were asked to avoid strenuous exercise and consumption of alcohol or medication during the previous 2 days. Interview questionnaires were used to check for compliance when participants arrived to the laboratory. The participants were asked to follow a hydration protocol and to come the laboratory every morning during 1 week to record their weights in order to ensure euhydration status. The average body mass during the last 4 days was considered as euhydration status if the standard deviation was < 150 g. Three participants did not fulfill the inclusion criteria of achieving a VO_2max_ above 50 ml/kg/min and 3 participants were not able to run for at least 40 min in one of the 2 trials days. The final study sample therefore included 16 individuals, their features and body composition are described in Table 2.

**Table 2:** Body composition and physical characteristics of the participants at baseline

Body composition	Mean ± SD	Minimum	Maximum
Age (years)	21.1 ± 1.4	19	24
Weight (kg)	74.1 ± 6.5	60	85.2
Height (m)	1.78 ± 0.04	1.7	1.86
Mesomorph somatotype	5.5 ± 0.9	4	7.3
Endomorph somatotype	2.1 ± 0.6	0.8	3.2
Ectomorph somatotype	2.4 ± 0.8	1.1	3.6
Waist to hip ratio	0.8 ± 0.02	0.8	0.84
Waist to hip index	0.44 ± 0.02	0.44	0.47
Fat mass percentage (DEXA) (%)	14 ± 5	6	22
Fat free mass percentage (DEXA) (%)	86 ± 5	78	94
Maximal oxygen uptake (mL/kg/min)	56 ± 4	49	63
Maximal heart rate (beat per min)	196 ± 7	183	206
Maximal Aerobic Speed (km/h)	16 ± 1	14	18

*SD* standard deviation

Participants performed two identical trials, 3 weeks apart, with different rehydration in a hot laboratory setting. Temperature (35 ± 1 °C) and relative humidity (60 ± 2 %) in the laboratory were kept constant with an electric heating system and continuously checked using a portable digital weather tracker (Radio Controlled, Oregon Scientific). The exercise trial consisted of treadmill running for up to 1 h at 60 % VO_2max_ (see below). After exercise, they were offered to drink either mineral water *ad libitum* or up to 660 ml of standard beer (4.5 %) and then water *ad libitum*. Rehydration strategies were randomly assigned and then swapped for the second trial, so that each participant was his own control. A set of measurements including body composition, hematological and serum parameters, fluid balance, urine excretion (both composition and total urine volume), hormones, markers of muscular damage and inflammation parameters was assessed before exercise, immediately after exercise, and after 2 h of rehydration phase.

### Running protocol

When the participants arrived to the laboratory they were asked to void all urine before measuring body mass in order to determine euhydration status. Differences in body mass compared to body mass in the previous week were not found (<150 ml) and the urine color was considered adequate within the lighter color of the scale [21]. The running protocol was performed on a treadmill (h/p/cosmos®, Nussdorf-Traunstein, Germany), starting with 5 min of warming-up at 40 % of the individual MAS, followed by up to 60 min at 60 % MAS (average speed: 9.6 ± 0.6 km/h), and 5 min at 30 % MAS for cooling down. Each participant provided a rating of his own thirst perception before and after the running protocol, using a category scale ranging from one (“not thirsty”) to ten (“very, very thirsty”), as indicated elsewhere [22]. Seven participants completed the 60-min running time, while the rest of the sample (nine) performed between 40 and 59 min (average time: 52.4 ± 7.6 min). The rate of perceived exertion (RPE) was evaluated immediately before and after the running protocol using a standardized Borg scale board [23]. The exercise protocol was perceived as hard and increased from 8 to 18 in RPE (*p* < 0.001). Maximal heart rate percentage increased from 60 % to 95 % (*p* < 0.001). A fan was used in order to facilitate sweat evaporation and thermoregulation, and for comfort reasons. Participants were advised to run wearing only shorts.

### Rehydration phase

The rehydration phase was conducted by one of two different rehydration strategies, randomly assigned before the first trial. Participants drank either standard beer (4.5 %) up to maximum 660 ml followed by mineral water *ad libitum* (intervention) or mineral water alone *ad libitum* (control). In the beer trial, participants’ were able to drink less than the 660 ml of beer available prior to consuming water *ad libitum*, however, all participants voluntary drank 660 ml of beer before continue with water. On the second trial, participants who had received the intervention on the first trial were given the control treatment, and vice versa. During the rehydration phase, the participants were asked to sit quietly in the laboratory and were allowed to drink the beverages on demand during a 2 h period. A researcher was in the room to serve liquids (6 °C) in doses of 200 ml on demand (glass beaker) and total fluid intake (ml) and time required were registered. Despite the ACSM guidelines prescribe a rehydration volume intake of 150 % of body mass loss [1], the current study include an *ad libitum* intake, however, due to the alcohol content beer was limited up to maximum 660 mL. Moreover, the thirst scale was used during the rehydration protocol to ensure that *ad libitum* rehydration was enough to calm the thirst.

### Set of measurements

The set of measurements included several dimensions such as body composition, hematologic and serum profile, fluid balance and urine excretion in order to explore the consequences at different levels (for detailed description of the techniques, methods and complementary parameters, see Additional file 1: Tables S1 and S2). Briefly, body mass (kg) and height (cm) were measured with a standard balance beam incorporating a stadiometer (SECA, Vogel & Halke GmbH & CO. Hamburg, Germany; precision of 0.1 kg and 0.1 cm) with participants in underwear and barefoot [24]. In addition, fat free mass and fat mass were measured by full body dual energy X-ray absorptiometry (DXA) using a Norland XR- 46 scanner (Norland, Fort Atkinson, WI) located in the exercise laboratory. Blood and urine parameters were also assessed. A 5 mL blood sample was taken three times by puncture of the cubital vein with minimum stasis and participants seated in comfortable position. The first blood sample was taken just before the exercise bout, after 8-h fasting plus one 1-h rest period; the second and third blood samples were taken immediately after exercise and rehydration, respectively. The samples were immediately aliquoted and stored at −80 °C until determination. The complete list of biological measurements included hematocrit (%), hemoglobin, mean corpuscular volume (MCV [fL]), changes in plasma volume (%), iron (g/dL), urea (mg/dL), creatinine (mg/dL), sodium (mEq/L) and potassium (mEq/L). Changes in plasma volume (%) were calculated [25]. The total urine volume (mL) was registered during the rehydration period (2 h) and during the last set of measurements (1.5 h) when participants needed to void, thus, the urine excretion rate (mL/min) was calculated from these variables. Urinary osmolality (mOsm/kg), urea (mg/dL), creatinine (mg/dL), uric acid (mg/dL), potassium (mEq/L), sodium (mEq/L), calcium (mg/dL), phosphorus (mg/dL), chlorine (mEq/L) and magnesium (mg/dL) were measured. The absolute excreted values were derived from the urine concentrations values and the total urine volume excreted in each trial.

### Statistical analyses

Predictive Analytics SoftWare (PASW, version 18; SPSS Inc., Chicago, IL, USA) was used to perform the analyses. Statistical significance was set at *p* < 0.05. The data are presented as mean ± standard deviation (SD) or 95 % confidence intervals as appropriate. The changes across the 3 points of measurements for all the variables were analyzed using the general linear model (GLM) repeated measure adjusting for treatment, group and order, with Bonferroni pairwise *post-hoc* comparisons. Relative increments between the two post-exercise measurement points (B and C) were calculated with the formula: Δ = (C-B)/B. The differences between the relative increments in each rehydration strategy were examined using the GLM univariate analysis after adjusting for treatment. All the residuals showed a satisfactory pattern.

## Results

### Running protocol

After the running protocol, mean heart rate, thirst scores and RPE values were significantly increased (*p* < 0.001) compared with pre-exercise (115 ± 6.5 *vs.*185 ± 7.5 beats per min; 2 ± 1 *vs.* 9 ± 1.3 thirst scores and 8 ± 1.2 *vs.*18 ± 1.8 point for RPE, respectively). Post-exercise, after 5 min resting, mean heart rate and RPE significantly decreased (185 ± 7.5 *vs.* 144 ± 5.1 beats per min and 18 ± 1.8 *vs.* 10 ± 1 point for RPE) (*p* < 0.001). There were no significant differences between the 2 days of evaluation. The exercise-induced dehydration was around 2.5 % (W, 2.4 % ± 0.3 %, BW, 2.3 % ± 0.1) of the body mass in both trials.

### Body composition, hematologic and serum parameters

Table 3 shows pre-exercise, post-exercise and post-rehydration levels of body composition, hematologic and serum parameters. Body mass, fat free mass, plasma volume and MCV significantly decreased, while iron, urea and creatinine significantly increased after exercise in both tests (*p* < 0.05). After rehydration, body mass and fat free mass significantly increased (*p* < 0.001), although body mass still remained under the baseline levels with both rehydration strategies (*p* < 0.001). Particularly, the average weight balance between post-rehydration and pre-exercise was minus 0.7 kg (<1 % of initial body mass) with both strategies. Similarly, urea and creatinine decreased significantly after rehydration (*p* < 0.05), with urea remaining significantly higher than baseline levels with the control (water alone) strategy (*p* < 0.05). Moreover, potassium levels also decreased after rehydration with both strategies (*p* < 0.05), despite not having changed during the exercise bouts. No differences between rehydration strategies for any of the variables were found at the end point (all *p* > 0.2, except for urea, *p* = 0.09).

**Table 3:** Body composition, hematologic and serum parameters through the whole protocol

	Pre-exercise	Post-exercise		Post-rehydration	*p*-value^*a*^
Weight (kg)	74.2 ± 6.5	72.4 ± 6.3^*d*^	Water	73.5 ± 6.5^*gj*^	*p* = 0.23
	74.3 ± 6.8	72.6 ± 6.7^*d*^	Beer + water	73.6 ± 6.9^*gj*^	
Fat free mass (DXA) (kg)	57.9 ± 4.5	56.6 ± 4.5^*d*^	Water	57.4 ± 4.6^*g*^	*p* = 0.40
	58.1 ± 4.9	56.9 ± 4.7^*d*^	Beer + water	57.5 ± 4.5^*g*^	
Fat mass (DXA) (kg)	10.5 ± 3.8	10.0 ± 3.6	Water	10.3 ± 3.6	*p* = 0.32
	10.5 ± 3.7	10.0 ± 3.3	Beer + water	10.1 ± 3.7	
Hematocrit (%)	45.7 ± 3.0	46.6 ± 2.5	Water	45.5 ± 2.3	*p* = 0.45
	45.1 ± 2.9	45.6 ± 2.2	Beer + water	45.0 ± 2.1	
Plasma volume changes (%)		−5.3 (−8.3;−0.1)^*b*^	Water	3.5 (0.9;9.3)	*p* = 0.44
		−5.1 (−5.7; 0.7)^*b*^	Beer + water	3.3 (0.5;7.9)	
MCV (fL)	87.7 ± 3.0	87.4 ± 2.8^*b*^	Water	87.5 ± 3.0	*p* = 0.40
	88.1 ± 3.4	87.8 ± 3.4^*c*^	Beer + water	87.7 ± 3.5	
Iron (g/dL)	95.7 ± 27.3	125.0 ± 31.6^*d*^	Water	122.9 ± 29.9	*p* = 0.43
	92.6 ± 32.0	114.6 ± 32.5^*b*^	Beer + water	108.0 ± 30.5	
Urea (mg/dL)	40 ± 6	47 ± 6^*d*^	Water	44 ± 4^*fi*^	*p* = 0.09
	39 ± 8	45 ± 7^*d*^	Beer + water	40 ± 8^*g*^	
Creatinine (mg/dL)	1.2 ± 0.1	1.3 ± 0.1^*d*^	Water	1.2 ± 0.1^*g*^	*p* = 0.25
	1.2 ± 0.1	1.3 ± 0.1^*d*^	Beer + water	1.2 ± 0.1^*e*^	
Sodium (mEq/L)	138 ± 2	138 ± 2	Water	137 ± 3	*p* = 0.95
	138 ± 2	139 ± 3	Beer + water	137 ± 2	
Potassium (mEq/L)	4.6 ± 0.4	4.7 ± 0.4	Water	4.2 ± 0.4^*gi*^	*p* = 0.28
	4.7 ± 0.4	4.7 ± 0.3	Beer + water	4.3 ± 0.3^*gh*^	

^*a*^differences between rehydration strategies

Post- vs Pre-exercise ^*b*^ *p* ≤ 0.05; ^*c*^ *p* ≤ 0.01; ^*d*^ *p* ≤ 0.001. Post-rehydration vs Post-exercise ^*e*^ *p* ≤ 0.05; ^*f*^ *p* ≤ 0.01; ^*g*^ *p* ≤ 0.001. Post-rehydration vs Pre-exercise ^*h*^ *p* ≤ 0.05; ^*i*^ *p* ≤ 0.001

*DXA* dual energy X-ray absorptiometry, *MCV* mean corpuscular volume

### Fluid balance and urine excretion

Table 4 details the status of fluid balance and urine excretion. None of the parameters analyzed differed significantly between rehydration strategies. Voluntary total fluid intake was approximately 90 % of the body mass lost after exercise; after taking into account the total urine volume, the fluid balance resulted in approximately 80 % of the body mass lost. The time spent for voluntary total fluid intake ranged from 20 to 40 min. Thirst scores after rehydration protocol were 1 for all the participants in both trials.

**Table 4:** Fluid balance and urine excretion

		Post-rehydration	*P* value^*a*^
Total fluid intake (mL)	Water	1644 ± 620	*p* = 0.91
	Beer + water	1620 ± 587	
Total urine volume (mL)	Water	223 ± 245	*p* = 0.70
	Beer + water	281 ± 374	
Fluid balance (mL)	Water	1429 ± 490	*p* = 0.51
	Beer + water	1329 ± 350	
Urinary excretion rate (mL/min)	Water	1.86 ± 2.04	*p* = 0.66
	Beer + water	2.34 ± 3.12	
Urinary osmolality (mOsm/kg)	Water	681.50 ± 181.04	*p* = 0.28
	Beer + water	587.17 ± 252.23	
Urea (g)^b^	Water	3.4 ± 2.7	*p* = 0.17
	Beer + water	2.5 ± 1.1	
Creatinine (g)^b^	Water	0.30 ± 0.16	*p* = 0.14
	Beer + water	0.25 ± 0.15	
Uric Acid (mg)^b^	Water	40 ± 42	*p* = 0.88
	Beer + water	39 ± 43	
Potassium (mEq)^b^	Water	9.80 ± 3.70	*p* = 0.15
	Beer + water	8.33 ± 3.45	
Sodium (mEq)^b^	Water	12 ± 8	*p* = 0.67
	Beer + water	13 ± 7	
Calcium (mg)^b^	Water	20 ± 16	*p* = 0.63
	Beer + water	20 ± 10	
Phosphorus (mg)^b^	Water	69 ± 82	*p* = 0.27
	Beer + water	45 ± 26	
Chlorine (mEq)^b^	Water	153 ± 98	*p* = 0.84
	Beer + water	154 ± 78	
Magnesium (mg)^b^	Water	8 ± 4	*p* = 0.50
	Beer + water	10 ± 5	

^*a*^differences between rehydration strategies

^b^absolute urinary excretion

Results for complementary parameters are showed in Additional file 1.

## Discussion

The main findings from our study show that restoration of markers of hydration after a dehydrating bout of exercise in the heat is not negatively influenced by the acute intake of a moderate amount of beer (up to 660 mL) as part of the rehydration strategy. The present study also shows that the voluntary fluid intake in the 2 h following exercise and based on thirst sensation is not enough to completely recover the initial hydration status and this is neither influenced by beer intake or its palatability.

Although there are few studies analyzing the role of moderate alcohol intake on hydration status after exercise, our results partially concur with previous studies regarding the lack of negative effects of moderate alcohol intake after exercise [13–15]; however, differences in methodology, exercise protocols and alcohol usage hamper the comparison among studies. Shirreffs and Maughan (1997) studied the effect of the consumption of four beverages with different alcohol concentrations (0, 1, 2 and 4 %) on the restoration of fluid and electrolyte balance after exercise-induced dehydration (loss of 2 % body mass, *n* = 6). The average volume consumed was 2212 mL, well above the amount provided in our study (up to 660 mL). They found that there was no difference in body water content after rehydration with beverage without alcohol, or containing 1 or 2 % of alcohol. By contrast, 4 % alcohol drink increased urine output and delayed the recovery process. In our study, no differences were found for any of the parameters assessed such as total urine volume, electrolyte excretion or body mass recovery when participants drank up to 660 mL of 4.5 % alcohol beer as part of the rehydration strategy, compared with drinking only mineral water. However, the final concentration of alcohol in relation to total fluid intake in our study (1.8 %) was similar to that reported by Shirreffs and Maughan (1997) (2 %), and the total amount of alcohol ingested was even lower (~24 g *vs.* 35 g in Shirreffs and Maughan [1997]). An important difference between our study and that of Shirreffs’ is the use of regular beer, which is a commonly consumed beverage, instead of a prepared solution with alcohol. Recently, Desbrow et al. [15] analyzed the role of adding sodium in two type of beers with different alcohol concentrations and they found similar conclusions that Shirreffs and Maughan (1997) regarding the lower urine output with ≤2 % of alcohol concentration [15]. Despite that on both studies [13, 15] a higher volume and total amount of alcohol intake were used in the rehydration protocol and consequently their participants registered higher total volume of urine output, we concur with them in the lack of negative influence on hydration when the final alcohol concentration is around 2 % (1.8 % in the present study).

Hobson and Maughan (2010) performed an interesting study in 12 males aimed to examine the effect of beer intake (4 % alcohol) on urine production in euhydrated and hypohydrated individuals. With this purpose, the participants completed an intermittent cycling protocol in hot and humid conditions (35 °C and 68 %, respectively). On the following morning the participants were provided with 1000 mL of alcohol-free beer or 960 ml of this alcohol-free beer with 40 ml of ethanol added. They observed that ingestion of alcohol (4 %) did not cause differences in the urine volume between alcohol and non-alcohol beer when hypohydrated, but there were differences when euhydrated. The results in hypohydrated participants are in agreement with our study, as neither total urine volume, urine solutes nor plasma volume were different when using alcohol beer (4.5 %) plus water or water alone in dehydrated participants. However, important differences exist between the exercise protocols for inducing dehydration of the above-mentioned studies [13–15], when compared with the current study. The main difference is that those authors used exercise specifically for achieving the dehydration status, while in our study we aimed to recreate a real life situation such as a short bout of exercise under hot conditions that induces dehydration, followed by moderate intake of beer (not prepared solution). Other studies have analyzed the effect of alcohol on hematological parameters and hydration status using high alcohol intakes, which constitutes a totally different research paradigm [12, 26].

The findings and information on hormones, muscular damage, and stress and inflammation parameters from the current study (Additional file 1) add a new dimension to the classical variables assessed in previous studies [13, 14], although these aspects have been partially addressed in recent studies on muscular performance but using different doses of alcohol (high intake) and methodologies [10, 11, 27]. Briefly, In our study the consumption of a moderate intake of beer (up to 660 ml) plus water did not influence the normal recovery of several indicators of physiological stress and inflammation compared with a rehydration with water alone in participants dehydrated after exercise in the heat. These findings add new insights on the dose-response between beer intake and recovery processes.

The reasons why moderate alcohol intake does not substantially affect diuresis or hydration status in dehydrated participants are not totally clear, but some hypotheses have been suggested. Alcohol has classically been shown to exert its diuretic action by the inhibitory effect of ethanol on vasopressin release from the posterior pituitary gland [28]. However, with perspiration (and subsequent decrease in plasma volume and increase in plasma osmolality), a counteracting reduction in urine production could also be expected as a result of stimulation of vasopressin release. Which of these two opposite mechanisms will be the dominant might depend on the level of dehydration [9, 14], but could also depend on the effectiveness, lifespan of alcohol in the body or the type of beverage/nutrients included. Thus, it could be expected that the diuretic response to moderate alcohol intake would be blunted as the level of dehydration increased [29]. Additionally, the human body has the ability to metabolize moderate quantities of alcohol through the family of enzymes called alcohol dehydrogenases [30]. Unfortunately, our study was not able to assess vasopressin, aldosterone or alcohol dehydrogenases in order to better understand the mechanisms underlying our results.

To the best of our knowledge this is the first study examining a practical and widespread use such as the inclusion of a moderate consumption of beer as part of the rehydration strategy after exercise. Moreover, the use of a naturally-fermented and habitual beverage rather than prepared solutions, the implementation of a common exercise protocol among amateur sport participants and the inclusion of an extensive set of precise measurements are strong points of this study. However, the present research has some limitations. First, due to the type of beverage used, a double-blind design was not possible. Secondly, the lack of information regarding vasopressin, aldosterone, alcohol dehydrogenases or blood alcohol, partially limits the interpretation of the results; however, other authors assessed these parameters in similar studies, obtaining results coherent with our findings [13, 14]. The fact that the total amount of fluid consumed was not fixed (a priori) between trials could constitute a limitation; however, we aimed to provide information about the voluntary total fluid drank when beer is included in the rehydration (up to 660 ml) rather than high beer consumption. Moreover, we were not able to take blood samples hourly or after 48 h which would allow to better knowledge the evolution of these measurements. Further research is required to provide more insight into the effects of different rehydration strategies and using different types of beverages, including alcohol on recovery.

## Conclusion

Our observations suggest that after exercise in the heat, and subsequent water losses, the acute intake of a moderate amount of beer (up to 660 ml) has no deleterious effects on markers of hydration neither on indicators of physiological stress recuperation in young healthy, physically active, male individuals. Taken together these findings we could advise those who regularly consume beer after sport or are physically active in the heat that a moderate intake would not alter the recuperation process. Nevertheless, high alcohol intake should not be recommended as the physiological and health consequences could be dangerous.

## Additional file

Additional file 1:
**Additional methodological and result issues.**

## Abbreviations

ACSM: American College of Sport Medicine
BM: Body mass
BW: Beer followed by water
CPK: Creatine phosphokinase
CRP: C-reactive protein
CSIC: Committee of the Spanish National Research Council
DXA: Dual energy X-ray absorptiometry
GLM: General linear model
HGH: Growth hormone
IgA: Immunoglobulin A
LDH: Lactate dehydrogenase
MAS: Maximal aerobic capacity
MCV: Mean corpuscular volume
RPE: Rating of perceived exertion
SD: Standard deviation
W: Water
