# Supplementary material for: Effects of a moderate intake of beer on markers of hydration after exercise in the heat: a crossover study
Source: J Int Soc Sports Nutr. 2015 Jun 6;12:26. doi: 10.1186/s12970-015-0088-5 (PMC4459073; doi:10.1186/s12970-015-0088-5)
Supplement: Additional file 1: — Additional methodological and result issues. [file 12970_2015_88_MOESM1_ESM.doc]

| **Table S1.** Description of methods, techniques and materials used for blood sample and urine measurements. | |
| --- | --- |
| **Measurements** | **Methods, techniques and materials** |
| *Blood Sample* |  |
| Hematocrit (%) | whole blood collected into EDTA-K3E-Vacutainer (BD) and automated analyser (Technicon H1, Bayer, Tarrytown, NY) |
| Mean corpuscular volume (fL) | whole blood collected into EDTA-K3E-Vacutainer (BD) and automated analyser (Technicon H1, Bayer, Tarrytown, NY) |
| Changes in plasma volume | The Synchron LX system using a Beckman analyzer (Fullerton, USA). |
| Iron (g/dL) | Enzymatic colorimetry using an auto-analyzer Olympus AU 2700 (Olympus, center Valley, PA USA). |
| Urea (mg/dL) | Enzymatic colorimetry using an auto-analyzer Olympus AU 2700 (Olympus, center Valley, PA USA). |
| Creatinine (mg/dL) | Enzymatic colorimetry using an auto-analyzer Olympus AU 2700 (Olympus, center Valley, PA USA). |
| Sodium (mEq/L) | Enzymatic colorimetry using an auto-analyzer Olympus AU 2700 (Olympus, center Valley, PA USA). |
| Potassium (mEq/L) | Enzymatic colorimetry using an auto-analyzer Olympus AU 2700 (Olympus, center Valley, PA USA). |
| Glucose (mg/dL) | Enzymatic colorimetry using an auto-analyzer Olympus AU 2700 (Olympus, center Valley, PA USA). |
| Insulin (μU/mL) | Chemiluminescence (EIA) (Advia Centaur, Siemens, Derfield, USA) |
| Cortisol (μg/dL) | Chemiluminescence (EIA) (Advia Centaur, Siemens, Derfield, USA) |
| Growth hormone (ng/mL) | Chemiluminescence (EIA) (Inmulite 2000, Siemens, Derfield, USA). |
| Homocysteine (µmol/L) | Chemiluminescence (EIA) (Inmulite 2000, Siemens, Derfield, USA). |
| Lactate dehydrogenase (U/L) | Auto-analyzer (Technicon RA-500, Bayer, Milan, Italy). |
| Creatine phosphokinase (U/L) | Auto-analyzer (Technicon RA-500, Bayer, Milan, Italy). |
| C-reactive protein (mg/L) | Nephelometry |
| Salivary IgA (mg/L) | Nephelometry |
|  |  |
| *Urine volume and composition* |  |
| Urinary osmolality (mOsm/kg) | by osmometry using an osmometer (Fiske 210, Nordwood, MA USA) |
| Urea (mg/dL) | by enzymatic colorimetry (auto-analyzer Olympus AU 2700) |
| Creatinine (mg/dL) | by enzymatic colorimetry (auto-analyzer Olympus AU 2700) |
| Uric acid (mg/dL) | by enzymatic colorimetry (auto-analyzer Olympus AU 2700) |
| Potassium (mEq/L) | by the selective electrode technique (auto-analyzer Olympus AU 2700) |
| Sodium (mEq/L) | by the selective electrode technique (auto-analyzer Olympus AU 2700) |
| Calcium (mg/dL) | by enzymatic colorimetry (auto-analyzer Olympus AU 2700) |
| Phosphorus (mg/dL) | by enzymatic colorimetry (auto-analyzer Olympus AU 2700) |
| Chlorine (mEq/L) | by the selective electrode technique (auto-analyzer Olympus AU 2700) |
| Magnesium (mg/dL) | by enzymatic colorimetry (auto-analyzer Olympus AU 2700) |

**Set of additional measurements**

Additionally, glucose (mg/dL), insulin (μU/mL), cortisol (μg/dL), growth hormone (HGH [ng/mL]), homocysteine (µmol/L), lactate dehydrogenase (LDH [U/L]), creatine phosphokinase (CPK [U/L]), C-reactive protein (CRP [mg/L]) and salivary immunoglobulin A (IgA [mg/L]) were measured.

**Additional results**

*Hormones, muscular damage and inflammation parameters*

The levels in relevant hormones, markers of muscular damage and inflammation parameters by rehydration strategies are showed in additional table 1. The glucose/insulin ratio and the levels of cortisol, HGH, LDH, CPK and salivary IgA were significantly increased through both exercise trials (p<0.05). After rehydration, glucose, cortisol, HGH and salivary IgA decreased significantly with both strategies, leaving glucose/insulin ratio and cortisol levels below baseline values (p<0.05). On the contrary, LDH remained significantly higher than baseline after rehydration with both strategies (p<0.05). Insulin levels showed a decreasing trend after exercise that progressed during rehydration, reaching statistical significance with the two strategies (p<0.05). No differences between rehydration strategies for any variable were found at the end point (after rehydration).

| **Table S2.** Levels of hormones and markers of muscular damage and inflammation through the whole protocol | | | | | |
| --- | --- | --- | --- | --- | --- |
|  | **Pre-exercise** | **Post-exercise** |  | **Post-rehydration** | **P value*^a^*** |
| Glucose (mg/dL) | 82 ± 13 | 95 ± 8 *^c^* | Water | 80 ± 6 *^g^* | p = 0.51 |
|  | 87 ± 14 | 89 ± 8 | Beer + water | 76 ± 10 *^g i^* |  |
| Insulin (μU/mL) | 18.1 ± 11.2 | 10.2 ± 8.4 | Water | 9.7 ± 7.6 *^h^* | p = 0.42 |
|  | 22.7 ± 14.2 | 14.4 ± 21.5 | Beer + water | 8.3 ± 3.5 *^i^* |  |
| Glucose/insulin ratio | 6.0 ± 3.0 | 12.4 ± 5.1 *^d^* | Water | 11.0 ± 4.8 *^i^* | p = 0.35 |
|  | 5.3 ± 3.0 | 11.0 ± 5.2 *^c^* | Beer + water | 10.2 ± 3.9 *^j^* |  |
| Cortisol (μg/dL) | 20.4 ± 4.7 | 28.1 ± 8.9 *^d^* | Water | 10.7 ± 3.4 *^g j^* | p = 0.53 |
|  | 19.0 ± 4.9 | 23.6 ± 6.4*^c^* | Beer + water | 10.2 ± 6.0*^g i^* |  |
| HGH (ng/mL) | 0.6 ± 0.8 | 6.5 ± 7.4 *^b^* | Water | 0.9 ± 1.0 *^e^* | p = 0.17 |
|  | 0.5 ± 0.9 | 4.6 ± 5.0 *^b^* | Beer + water | 0.3 ± 0.3 *^f^* |  |
| LDH (U/L) | 353 ± 66 | 375 ± 60 *^b^* | Water | 396 ± 59 *^h^* | p = 0.13 |
|  | 336 ± 64 | 389 ± 50 *^c^* | Beer + water | 381 ± 44 *^h^* |  |
| CPK (U/L) | 201 ± 191 | 246 ± 206 *^d^* | Water | 225 ± 186 | p = 0.78 |
|  | 280 ± 353 | 339 ± 402 *^c^* | Beer + water | 309 ± 367 |  |
| Salivary IgA (mg/L) | 10.5 ± 4.5 | 19.6 ± 12.1 *^b^* | Water | 8.3 ± 2.9 *^e^* | p = 0.44 |
|  | 11.9 ± 5.1 | 21.4 ± 11.4 *^b^* | Beer + water | 8.4 ± 5.2 *^e h^* |  |
| Homocysteine (µmol/L) | 11.2 ± 7.1 | 12.7 ± 8.9 | Water | 14.0 ± 11.6 | p = 0.84 |
|  | 10.7 ± 5.3 | 12.4 ± 7.3 | Beer + water | 13.1 ± 6.2 |  |
| C-Reactive Protein (mg/L) | 1.19 ± 1.49 | 1.22 ± 1.57 | Water | 1.16 ± 1.47 | p = 0.31 |
|  | 1.11 ± 1.15 | 1.11 ± 1.23 | Beer + water | 1.06 ± 1.17 |  |
| *^a^*differences between rehydration strategies. | | | | | |
| Post- vs Pre-exercise *^b^*p≤ 0.05; *^c^*p≤ 0.01; *^d^*p≤ 0.001. Post-rehydration vs Post-exercise *^e^*p≤ 0.05; *^f^* p≤ 0.01; *^g^*p≤ 0.001. Post-rehydration vs Pre-exercise *^h^*p≤ 0.05; *^i^*p≤ 0.01; *^j^*p≤ 0.001 | | | | | |
| CPK, creatine phosphokinase; HGH, human growth hormone; IgA, Immunoglobulin A; LDH, lactate dehydrogenase | | | | | |
